# Perspectives on Outpatient Delivery of Bispecific T-Cell Engager Therapies for Multiple Myeloma

**DOI:** 10.3390/curroncol32040238

**Published:** 2025-04-18

**Authors:** Andrée-Anne Pelland, Mathilde Dumas, Émilie Lemieux-Blanchard, Richard LeBlanc, Julie Côté, Jean-Samuel Boudreault, Dominic Duquette, Rayan Kaedbey, Marc Lalancette, Frédéric Larose, Anna Nikonova, Michel Pavic, April Shamy, Jean Roy, Michael Sebag, Sabrina Trudel, Jean-Sébastien Claveau

**Affiliations:** 1 BC Cancer, Vancouver, BC V5Z 4E6, Canada; andree-anne.pelland.med@ssss.gouv.qc.ca; 2 Centre Hospitalier Universitaire de Québec (CHU), Québec, QC G1V 0E8, Canada; 3 Hôpital Maisonneuve-Rosemont, Montréal, QC H1T 2M4, Canada; 4 Centre Hospitalier de l’Université de Montréal (CHUM), Montréal, QC H2X 3E4, Canada; 5 Hôpital du Sacré-Cœur de Montréal, Montréal, QC H4J 1C5, Canada; 6 Jewish General Hospital, Montréal, QC H3T 1E2, Canada; 7 Hôpital Hôtel-Dieu de Lévis, Lévis, QC G6V 3Z1, Canada; 8 McGill University Health Center (CUSM), Montréal, QC H3A 0G4, Canada; 9 Centre Hospitalier Universitaire de Sherbrooke (CHUS), Sherbrooke, QC J1H 5H3, Canada; 10 Hôpital Charles-Le Moyne, Greenfield Park, QC J4V 2H1, Canada

**Keywords:** bispecific antibodies, multiple myeloma, targeted immunotherapy, toxicity

## Abstract

In the past few years, a new promising therapy, called bispecific T-cell engager (TCE), has been developed and is now available in many countries for patients with relapsed or refractory multiple myeloma. T-cell engagers are associated with sustained efficacy and progression-free survival benefits in patients with heavily treated myeloma. However, complications such as cytokine release syndrome (CRS), immune effector cell-associated neurotoxicity syndrome (ICANS), and infections complicate their administration, particularly in remote centers. This review discusses the key requirements for delivering TCEs therapies, focusing on outpatient delivery. We also outline the primary acute and chronic complications of TCE therapy and their management.

## 1. Introduction

Multiple myeloma (MM) represents the third most common hematologic malignancy worldwide with almost 188,000 new cases diagnosed in 2022, according to the latest Global Cancer Observatory Report (GLOBOCAN) report [1]. In recent years, the overall survival (OS) of MM has improved with the emergence of novel therapies, even though patients invariably become refractory to all known treatments. Triple-class refractory MM represents a particular challenge and is characterized by the progression during or shortly after treatment with the three main classes of anti-MM therapies: anti-CD38 monoclonal antibodies; (MoAbs), proteasome inhibitors; (PIs), and immunomodulators (IMiDs). In triple-class refractory patients, the overall response rate (ORR) to subsequent therapies is poor at approximately 30%, based on the locoMMotion trial, with median progression-free survival (PFS) of 3 to 4 months and a median OS of approximately 12 months [2,3,4]. Emerging drugs with novel mechanisms of action, including bispecific T-cell engagers (TCEs), show promising outcomes for patients with relapsed or refractory multiple myeloma (RRMM).

TCEs are a class of immunotherapy that simultaneously bind to an antigen target at the surface of myeloma cells and to a T-cell, provoking T-cell activation and leading to tumor cell lysis via the release of granzymes, perforins, and cytokines. This activation is independent of the major histocompatibility complex (MHC) and from antigen-presenting cells [5,6]. Several plasma cell-specific antigens at the surface of myeloma cells have been studied as targets for TCEs, including B-cell maturation antigen (BCMA), G protein-coupled receptor, class C group 5 member D (GPRC5D), and Fc receptor-homolog 5 (FcRH5) [7,8,9,10,11].

As opposed to CAR-T cell therapy, TCEs are considered as an “off-the-shelf” therapy, which means they are readily available and do not require manufacturing and lymphodepletion before their administration. This provides a particular advantage for patients with rapidly progressive disease or those who are more frail. While no randomized clinical trials have directly compared the efficacy of these two approaches, TCEs are generally associated with a more favorable toxicity profile, which supports their use in outpatient settings. A recent meta-analysis reported an overall cytokine release syndrome (CRS) rate of 83% with CAR-T therapy versus 59% with TCEs, with grade 3 CRS occurring in 7% and 1% of patients, respectively [12].

## 2. Efficacity and Safety of Bispecific T-Cell Engager Therapies in Multiple Myeloma

### 2.1. Teclistamab

Teclistamab, a BCMA-CD3 bispecific T-cell engager, was first approved by the European Medicines Agency (EMA) in August 2022 for the treatment of patients who had previously received three or more therapies, by the US Food and Drug Administration (FDA) in October 2022, and by Health Canada in August 2023. The study leading to the approval of teclistamab was the phase 2 expansion trial MajesTEC-1, which enrolled 165 subjects being treated with teclistamab monotherapy. Patients had to have received at least three lines of therapy, including an IMiD agent, a PI, and an anti-CD38 MoAb. After a median of five prior lines of therapy, the ORR was 63.0%, with 39.4% achieving a complete response (CR) or better. The minimal residual disease (MRD) negativity rate among patients achieving a CR or better was 46%. After a median follow-up of 14.1 months, the median duration of response (DoR) was 18.4 months, and the median PFS was 11.3 months. Among common adverse effects, 72.1% of patients experienced cytokine release syndrome (CRS), 3.0% experienced immune effector cell-associated neurotoxicity (ICANS), and 76.4% experienced infections, including 12 patients (7.3%) that died from COVID-19 [13]. It is also important to note that in patients who have achieved and maintained a complete response (CR) or better for at least 6 months, the frequency of treatment can be extended to every 2 weeks.

### 2.2. Elranatamab

Elranatamab was FDA and EMA approved in August 2023, with Health Canada following suit in January 2024. Approval was granted on the results of the phase 2 MagnetisMM-3 study, which enrolled 123 patients with RRMM that were treated with fixed dose weekly elranatamab. The results reported an ORR of 61%, with a median DoR not reached, and a median PFS of 17.2 months. A CR or better was seen in 35.8% of patients, while MRD negativity was 89.7% in those eligible for analysis [14]. CRS occurred in 57.7% of patients, while ICANS was seen in 3.4%. All incidences of CRS and ICANS were grade 2 or lower. Infections occurred in 69.9% of subjects with 40.7% grade 3 or 4 events, and 6.5% experienced fatal infections. De-escalation of therapy to every 2 weeks was indicated for persistent responders (partial response (PR) or better lasting at least 2 months) after 6 months of therapy. Among responders who switched to every second week administration (n = 50), 80% maintained or improved their response at least 6 months after changing. The overall incidence of all grade 3 and 4 adverse events, including infectious AEs, decreased from 58.6% to 46.6% with de-escalation of treatment [15,16].

### 2.3. Talquetamab

Talquetamab was approved by the FDA and EMA in August 2023 and by Health Canada in May 2024. The phase 2 MonumenTAL-1 trial included 288 subjects who received talquetamab weekly (QW, n = 143) or every 2 weeks (Q2W, n = 145). Additionally, 51 subjects exposed to prior T-cell redirection therapy were randomized and received either dosing schedules. The QW, Q2W and previous T cell redirection cohorts were largely triple-class refractory at 74%, 69%, and 84%, respectively, with a median of 5–6 prior lines of treatment. In the prior T-cell redirection cohort, 71% had received CAR-T therapy, 35% had received a TCE, and 6% had received both. The ORRs were 74% (QW) and 73% (Q2W), with complete response or better in 23% (QW) and 22% (Q2W). In the prior T-cell redirection cohort, the ORR was 63% (53% ≥ VGPR). Median PFS was 7.5 months (QW), 11.9 (Q2W) months, and 5.1 months (prior T-cell redirection cohort). Common adverse effects included CRS in 79%, 75%, and 77% of patients, ICANS in 11%, 11%, and 3%, skin-related toxicity in 56%, 71%, and 69%, and dysgeusia in 50%, 48%, and 61%. Grade 3–4 infections occurred in 22%, 16%, and 26% of the QW, Q2W, and prior T-cell redirection cohorts, respectively [17,18].

## 3. Administration of T-Cell Engagers

Teclistamab, elranatamab, and talquetamab are all administrated subcutaneously and require premedication and step-up doses to mitigate the risk of acute toxicities (Table 1). According to the monograph, teclistamab is administrated at 15.5 mg/kg once weekly after two step-up doses of 0.06 mg/kg and 0.3 mg/kg separated by at least 48 h [13]. Based on a clinical trial, reducing the dose frequency to 1.5 mg/kg every 2 weeks (Q2W) is possible in patients who have achieved and maintained a complete response (CR) or better for a minimum of 6 months [19]. Elranatamab is administered at a fixed dose of 76 mg once weekly after two step-up doses of 12 mg and 32 mg separated by at least 48 h [15]. For patients that have achieved at least partial response at 2 months, with at least six 28-day cycles of elranatamab, injections can be given every 2 weeks [16]. Talquetamab is administered at 0.4 mg/kg once weekly or 0.8 mg/kg once every 2 weeks after two step-up doses of 0.01 mg/kg and 0.06 mg/kg separated by at least 48 h [17].

When a TCE therapy needs to be interrupted for a prolonged period (generally >4 weeks), it is advised to consider repeating the step-up dosing, according to product’s monograph, to avoid acute toxicities.

Recommended premedication, given during the dose escalation phases only, includes dexamethasone 16–20 mg PO/IV, loratadine 20 mg PO, and acetaminophen 650–1000 mg PO, administered 30 to 60 min prior to TCE injection. Subsequent premedication after the first full dose of the drug is at the discretion of clinician, but rarely needed.

### 3.1. Outpatient Bispecific T-Cell Engager Administration

**Figure 1:**
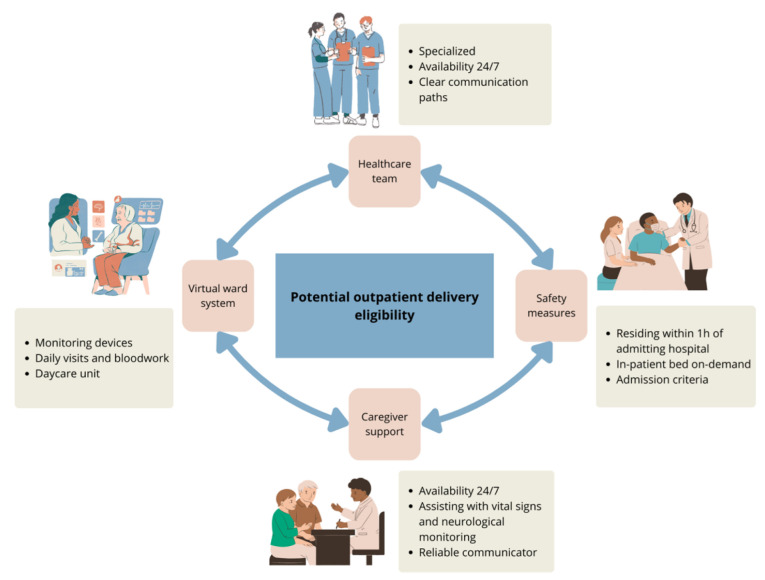
Key requirements for outpatient delivery of TCEs.

TCEs are among the most potent single-agent therapies for multiple myeloma ever developed. Their ready-to-use formulation and simple subcutaneous administration offer significant advantages, making them more accessible and easier to implement in community environments compared to cell-based therapies like CAR-T. However, their use in these settings introduces unique challenges not typically encountered with other treatments. One of the biggest caveats of out-patient administration is the need for ongoing monitoring of acute toxicities such as CRS and ICANS, which require rapid detection and intervention. Additionally, the increased risk of infectious complications and prolonged cytopenias demand heightened vigilance. These issues are further compounded by health systems with resource deficits, where limited infrastructures and staffing may hinder effective monitoring and management. Addressing these challenges requires careful planning and adequate resource allocation to ensure safe and efficient therapy delivery to all eligible patients (Figure 1). A recent review of 57 patients from the Mayo Clinic who received step-up dosing as outpatients showed that it was a safe and feasible option that potentially reduced healthcare resources utilization [20].

Patients in TCEs clinical trials were hospitalized for 48 h following each dose escalation and the initial full dose, although the product monographs do not specify in-patient administration. TCEs treatments require daily observation following dose escalation; however, with adequate support, they can be effectively managed through a virtual ward during the first 1–2 weeks of treatment. This model delivers hospital-level care at home using remote monitoring, digital technology, and coordinated medical oversight. To ensure the safe outpatient administration of TCEs, careful patient selection and continuous monitoring are essential, allowing for timely intervention in the case of CRS or ICANS. Patients should be capable of adhering to healthcare instructions, including self-administration of medications. Key safety measures for at home hospitalization include having a caregiver at home at all times for the first 2 weeks that can assist with frequent vital sign and neurological monitoring and maintaining communication with the specialized care team as needed. Additionally, patients should live within an hour of the admitting hospital and have access to day-care services and immediate on-demand inpatient bed availability in case of adverse events (Table A1).

For care teams, patient follow-up in the virtual ward can be facilitated by connected vital sign devices that trigger alerts if parameters become abnormal (e.g., Bluetooth-enabled blood pressure cuffs, pulse oximeters, and thermometers). Alternatively, vital signs can be assessed every four to eight hours using standard devices, with results communicated by the patient or their caregiver to the supervising medical team. In case of vital sign abnormality or new symptoms, there must be established communication paths and procedures to prevent emergency department visits. This includes a designated team member available 24/7 to communicate with the patient and their caregiver and that can advise on the management of the situation based on pre-determined institutional algorithms. Hence, establishing admission criteria to ensure prompt intervention for CRS, ICANS, infections, and other complications is essential for outpatient administration of TCEs (Table A2). A setup for direct admission to the oncology unit is crucial. Additionally, daily visits to a day-care unit for clinical assessment and bloodwork monitoring should be organized. This can also facilitate administration of IV medication, if needed, and permit surveillance of symptoms if admission is not initially warranted.

Discharge from the virtual ward can be considered when the subject is afebrile and has a normal immune effector cell-associated encephalopathy (ICE) score for at least 24–48 h after the first full dose (Table A3).

### 3.2. Mitigation of Cytokine Release Syndrome Risk

With the limitation of the available data, we believe that the prophylactic use of tocilizumab, an anti-IL6 receptor antibody, can be considered to reduce the incidence of CRS, but possibly not its severity. Based on small pilot studies, prophylactic tocilizumab (4 mg/kg IV) administered less than 4 h prior to the first TCE dose showed a reduction in grade 1 CRS occurrence, but not in higher-grade CRS [21]. The MajesTEC-1 prophylactic tocilizumab cohort showed a reduced rate of CRS following teclistamab administration of 25% compared to 72% in the non-prophylaxis cohort, and all events were noted to be of grade 1 or 2 [13]. Results from ongoing, but unreported, prospective studies on tociluzumab prophylaxis are expected. Special attention must be paid to the incidence of neutropenia and infection in subjects who receive tocilizumab or dexamethasone. Finally, dexamethasone 10–20 mg PO can be used as daily prophylaxis during step-up dosing or it can be considered as a “pill-in-the pocket” for in-home management of grade 1 CRS or ICANS.

## 4. Toxicity Management

TCEs are associated with acute and delayed toxicities. It is important that clinicians be aware of these complications and react to manage them appropriately in a timely manner. Acute reactions, within hours to the first few days, include CRS, ICANS, hepatotoxicity, and local site injection reactions. Long-term complications are mostly related to cytopenias and infections. To date, there are no signals concerning an increased risk of secondary malignancies.

### 4.1. Cytokine Release Syndrome

CRS is a systemic inflammatory reaction mediated by activated immune cells and their cytokines [22]. It has been reported in up to 87% of patients receiving a TCE. It usually begins with fever and non-specific constitutional symptoms such as fatigue and anorexia, but it can quickly escalate to life-threatening organ failure. Rapid recognition of CRS is crucial to ensure adequate treatment and minimize complications.

As fever is the cardinal manifestation of CRS, it is important to note that there is a significant overlap between CRS and infection or sepsis. A comprehensive infectious workup should be initiated in all febrile patients, especially in those who are neutropenic, and broad-spectrum antibiotics initiated when appropriate.

Infusion reactions can occur due to drug hypersensitivity. These can manifest with fever but are often accompanied by other manifestations including rash, dyspnea, hypotension, and/or gastrointestinal symptoms. They occur almost immediately following TCE infusion, while CRS generally occurs hours to days after infusion. The median time to CRS is 2 days after first infusion (range 1–6) and median duration is 2 days [13,15,17].

Grading of CRS is key to delivering appropriate treatment and, while different grading has been proposed, most centers use the American Society for Transplantation and Cellular Therapy (ASTCT) consensus grading system [23]. This method stratifies patients according to fever ≥ 38 °C, hypotension (with or without the use of vasopressor), and hypoxemia (with or without the necessity of differing oxygen-delivery methods) (Table 2). The cornerstones of CRS treatment are the prompt delivery of supportive care and the administration of tocilizumab and/or corticosteroids. It has been shown that early utilization of tocilizumab improves outcomes without compromising the efficacity of therapy [24].

Tocilizumab is administered intravenously at 8 mg/kg (maximum 800 mg) every 8 h for a maximum of three doses per 24 h. Alternatively, dexamethasone 10 mg po once can be given for grade 1 CRS. If CRS persists or recurs after 1–3 doses of tocilizumab, the use of dexamethasone given intravenously at 10 mg every 6 h should be considered. In patients with grade 3 or 4 CRS, tocilizumab, high-dose steroids (methylprednisone 1–2 g intravenously daily), and salvage CRS treatment (e.g., anakinra 100 mg BID subcutaneously twice daily, with a maximum dose of 48 mg/kg/day) must be considered in addition to transfer to a monitored care setting, such as an intensive care unit (Table 2). That said, few patients in MajesTEC-1, MagnetisMM-1, and MonumenTAL-1 needed repeated doses of tocilizumab (2.4%) or corticosteroids (4.6–8.5%) [13,15,17,25,26].

### 4.2. Immune Effector Cell-Associated Neurotoxicity Syndrome

ICANS is defined as central nervous system impairment after the infusion of a TCE therapy whose presentation can vary from a mild encephalopathy (inattention, word-finding difficulties) to more severe motor disturbances, convulsions, and coma [20]. Unlike with CAR-T cell therapy, the incidence of ICANS is low with TCEs, being reported in approximately 3% of patients receiving teclistamab or elranatamab and up to 10% of patients receiving talquetamab. All reported ICANS cases were of low grades (1–2) and occurred concomitantly or shortly after CRS. The median time to onset was 2.5 days, and median duration was 7 days. In the different studies, maximum reported time to onset of ICANS was 13 days after injection [13,15,17]. Other neurological events were also reported, with the most common being headaches (up to 20%). Ataxia, dizziness, and peripheral neuropathy were also described.

ICANS can mimic many other clinical conditions, and its diagnosis can be challenging. The immune effector cell-associated encephalopathy (ICE) scoring system (Table A4) provides objective and easy screening and grading of encephalopathy-associated symptoms [23]. This score is integrated into the ASTCT consensus grading for ICANS, which is necessary to determine adequate treatment (Table 3). Differential diagnosis of ICANS includes encephalopathy secondary to medication, infections, endocrinopathies, acute intracerebral ischemia or hemorrhage and, rarely, central nervous system infiltration by MM.

Neuroimaging should include, at least, a non-contrast brain CT to identify major bleeding or life-threatening cerebral edema, while a brain MRI should be considered to evaluate more subtle changes such as ischemic events or leptomeningeal processes. Depending on the state of consciousness and clinical suspicion, an electroencephalogram (EEG) and a lumbar puncture could be necessary. A neurology consultation must be included in the evaluation process. 

The mainstays of treatment of ICANS are glucocorticoids such as dexamethasone. Some patients with grade 1 might be observed but, considering the potential for rapid deterioration, we recommend giving dexamethasone 10 mg iv/po for one dose at first sign of ICANS and reassessing for the need of subsequent doses after 6 to 8 h. Patients with grade 2 ICANS usually require repeat doses of dexamethasone 10–20 mg every 6 h. Patients with grade 3–4 ICANS should be monitored in an intensive care setting and receive higher doses of corticosteroids (e.g., methylprednisolone 1000–2000 mg/kg IV) with or without anakinra 100 mg sc BID [27,28]. Levetiracetam 500 mg po BID should be initiated in patients with grade 3–4 ICANS to prevent convulsions. As there is a risk of worsening the ICANS, tocilizumab should not be given alone and only be considered if a concomitant CRS is present [26]. The majority of CRS and ICANS occur during the first cycle.

### 4.3. Infections

Infections are common in patients treated with TCEs and lead to frequent dose interruptions. The mechanisms making patients more vulnerable to infections are varied, but include frequent hypogammaglobulinemia, especially in patients receiving BCMA directed TCEs. TCEs may also increase the risk of infection by inducing T-cell exhaustion while subsequent T cell number and function decrease. Neutropenia is also frequent in the early weeks to months following TCE initiation [29,30]. Also, as BCMA are also present on mature plasma cells and on memory B-cells, destruction of those healthy cells with anti-BCMA drugs contributes to the prevalence of infections and its continuous risk with long-term therapy. TCE trials demonstrated rates of infections up to 70–75%, including grade 3 or 4 infections in up to 40–45% of patients [13,15,17]. Bacterial, viral, and fungal infections have all been described, the most common being respiratory infections and COVID-19. Opportunistic infections have also been reported, including cytomegalovirus (CMV), Epstein–Barr virus (EBV), pneumocystis jirovecii (PJP), adenovirus, parvovirus B19, human herpes virus 6 (HHV6), and progressive multifocal leukoencephalopathy.

The high frequency of infections has led to recommendations for baseline screening and prophylaxis [31,32]. Prior to initiating TCEs therapies, all patients should be screened for hepatitis B virus (HBV), hepatitis C virus (HCV), human immunodeficiency virus (HIV), Epstein–Barr virus (EBV), and carefully assessed for signs of active infections [26]. Results of these screening tests should not be awaited to begin therapy if there is a low likelihood of infection. Generally, screening for CMV reactivation is not recommended since the overall incidence remains low (no reactivation in 288 patients in MonumenTAL-1 [17], one reactivation in 55 patients in MagnetisMM-1 [15], and one reactivation in 165 patients in MajesTEC-1) [30]. If a patient presents symptoms of CMV infection and PCR-DNA shows ≥1000 copies/mL or if a positive biopsy is obtained, antiviral treatment is recommended. All patients with a potential to reactivate HBV (HBV core antibody positivity and/or HBV surface antigen) need to receive viral prophylaxis therapy (e.g., entecavir). Patients should be vaccinated, ideally prior to the initiation of TCEs, for COVID-19, influenza, herpes zoster virus (VZV), and pneumococcal pneumonia. There is no evidence about the best timing of vaccines for patients receiving TCEs. We would recommend following the ASCO guidelines suggesting that patients should have their vaccine schedule updated at least 2 weeks before therapy [33]. Prophylaxis against VZV and herpes simplex virus (HSV) should be initiated in all patients with valacyclovir 500 mg orally once daily. PJP prophylaxis is recommended for all patients with trimethoprim-sulfamethoxazole 800–160 mg one tablet three times weekly or adjusted for renal function or, in case of allergy or contraindication, atovaquone 1500 mg PO daily or nebulized pentamidine 300 mg every 4 weeks. Bacterial prophylaxis with levofloxacin 500 mg PO daily, moxifloxacin 400 mg PO daily, or doxycycline 200 mg PO daily can be considered in patients with prolonged neutropenia or previous history of recurrent bacterial infections [30]. In patients with prolonged neutropenia (<0.5 × 10^9^/L for at least 7 days) or prolonged corticosteroid therapy, fluconazole 400 mg PO daily should be considered until resolution of neutropenia or cessation of corticosteroids. Immunoglobin replacement (IVIG: 400 mg/kg every 2–4 weeks, or equivalent subcutaneous gammaglobulin preparations) should be initiated in patients with lower than 4 g/L of polyclonal IgG levels. Prophylactic IVIG use has been shown to reduce the incidence of grade 3–5 infections by 90% [8,26]. A summary of recommendations regarding infectious prophylaxis is included in Table 4.

Finally, during treatment, whenever active infection is diagnosed, the TCE therapy must be held until infection resolution.

### 4.4. Cytopenias

Cytopenias are a common adverse event of TCEs, with grade 3–4 neutropenia being reported in 26–70%, anemia in 33–51%, and thrombocytopenia in 13–29% [13,15,17]. The mechanism underlying cytopenias is poorly understood but could be attributed to poor bone marrow reserve in heavily pretreated patients, changes in the marrow microenvironment with impaired hematopoiesis secondary to cytokine release and TCE direct toxicity [32].

Management of cytopenias depends on their severity and consists of supportive care and dose delays in patients with grade 3 or more cytopenias, as per the International Myeloma Working Group guidelines (Table 5) [26]. Investigating other causes of cytopenias, such as iron and vitamin deficiency, myelodysplasia, and infections, is also primordial.

Finally, growth-colony stimulating factors (e.g., Filgrastim 300–480 mcg SC daily) should be considered in patients with neutropenia to keep neutrophil count above 1.0 × 10^9^/L.

### 4.5. Other Complications

Talquetamab, an anti-GPRC5D TCE, has a unique side effect profile attributable to an on-target, off-tumor effect. Besides being expressed by myeloma cells, GPRC5D is found in keratinized tissues such as hair follicles, skin, and lingual filiform papillae [34]. This results in dermatological toxicities in up to 70% of patients. The most common skin manifestations include palmoplantar keratoderma, xeroderma, and pruritus. Nail toxicities include Beau’s lines, onychodystrophy, and onycholysis [35,36]. Oral mucosal changes were also frequently described leading to xerostomia (70%), dysgeusia (50%), and weight loss (30%) [17,37]. Supportive measures are essential to optimize treatment tolerance and quality of life. For skin toxicities, moisturizing lotions, emollients, and topical steroids are encouraged. For oral symptoms, optimal mouth hygiene should be maintained, adequate hydration is essential, and saliva substitutes and corticosteroid mouthwash can be used. If weight loss or oral/swallowing symptoms persist or worsen despite adjunctive measures, dose interruption until improvement followed by dose reduction or dose frequency reduction may be required [38].

All TCEs are considered potentially teratogenic and risk mitigation strategies must be employed in the appropriate contexts. The products’ monographs advise to use effective contraception during treatment and for at least 5 months after the last dose for females and for 3 months after last dose for males.

## 5. Future Directions

In summary, TCEs are a promising new class of treatments for RRMM. Education about TCEs unique adverse events is primordial to ensure patients’ safety and optimal administration. To enable outpatient administration, including in community hospitals and remote centers, clinicians must be familiar with their distinct toxicities and specific management strategies. Tocilizumab prophylaxis also appears to be a promising and innovative method to facilitate the outpatient administration of TCEs. In addition to currently approved agents, several other TCEs—such as linvoseltamab (BCMA antibody), ABBV-383 (BCMA antibody), alnuctamab (bivalent BCMA antibody), forimtamig (GPRC5D antibody), ISB-1342 (CD38 antibody), and cevostamab (FcRH5 antibody)—are in development and may soon expand treatment options, allowing certain resistance mechanisms to be overcome and promoting a more individualized approach tailored to patient-specific characteristics. These expert recommendations will hopefully help clinicians to better supervise patients receiving TCEs therapies and help institutions create protocols to facilitate their outpatient delivery.

## Figures and Tables

**Table 1:** Proposition of doses escalation schedule.

Cycle, Day	Teclistamab (mg/kg)	Elranatamab (mg)	Talquetamab (mg/kg)
C1D1 ^†^	0.06	12	0.01
C1D3 ^†^	0.3	32	0.06
C1D5	1.5	-	0.4
C1D7	-	76	-
Cycle 2+	1.5 q 1 week *	C2-C24: 76 q 1 weeks C25 et +: 76 q 2 weeks **	0.4 q 1 week OR D10 onwards: 0.8 q 2 weeks

^†^ Minimum of 48 h between each step-up dose, * teclistamab: every (q) 2 weeks if complete response or better for more than 6 months, ** elranatamab: every (q) 2 weeks if partial response or better for more than 2 months.

**Table 2:** Grading and proposed management of cytokine release syndrome.

	Grade 1	Grade 2	Grade 3	Grade 4
Criteria	Fever ≥ 38 °C WITHOUT hypotension AND hypoxia	Fever ≥ 38 °C WITH hypotension not requiring vasopressors AND/OR hypoxia requiring ≤6 L/min O_2_	Fever ≥ 38 °C WITH hypotension requiring a vasopressor AND/OR hypoxia requiring >6 L/min O_2_	Fever ≥ 38 °C WITH hypotension requiring multiple vasopressors AND/OR hypoxia requiring positive pressure (CPAP, BiPAP, or mechanical ventilation)
Investigations and non-pharmaceutical	Blood cultures, chest X-ray, and urinalysis If outpatient, consider patient admission	Consider intensive care unit transfer Blood cultures, chest X-ray, and urine analysis Close monitoring of ferritin, fibrinogen, and INR Vital signs q 2–4 h Continuous cardiac monitoring	Intensive care unit transfer Hemocultures, chest X-ray, and urine analysis Close monitoring of ferritin, fibrinogen, and INR Serial vital signs Continuous cardiac monitoring	Intensive care unit transfer Hemocultures, pulmonary X-ray, and urine analysis Close monitoring of ferritin, fibrinogen, and INR Serial vital signs Continuous cardiac monitoring
Treatment	Supportive care (acetaminophen, broad spectrum antibiotics if neutropenic, IV fluids) Consider one dose of Tocilizumab 8 mg/kg IV or dexamethasone 10 mg if grade 1 or if fever persists for >24–48 h	Supportive care (acetaminophen, antibiotics, IV fluids) Tocilizumab 8 mg/kg IV and repeat q 8 h if no improvement (max. three doses) If no improvement, consider adding dexamethasone 10 mg IV q 6 h and/or anakinra 100 mg SC/IV q 12 h	Supportive care (acetaminophen, antibiotics, IV fluids, vasopressor) Tocilizumab 8 mg/kg IV and repeat q 8 h if (max. three doses) AND dexamethasone 10 mg IV q 6 h with anakinra 100 mg SC/IV q 12 h	Supportive care (acetaminophen, antibiotics, IV fluids, vasopressors) Tocilizumab 8 mg/kg IV and repeat q 8 h if (max. three doses) AND methylprednisolone 1000 mg IV DIE with anakinra 100 mg SC/IV q 12 h

INR: international normalized ratio; SC: subcutaneously; IV: intravenously; CPAP: continuous positive airway pressure; BiPAP: bilevel positive airway pressure.

**Table 3:** Grading and proposed management of immune effector cell-associated neurotoxicity syndrome.

	Grade 1	Grade 2	Grade 3	Grade 4
Criteria	ICE 7–9 Spontaneous awakening	ICE 3–6 Awakening on verbal stimulation	ICE 0–2 OR any clinical seizures, focal or generalized, that resolve rapidly OR non-convulsive seizures on EEG that resolve with intervention OR focal/local edema on neuroimaging	Unconscious patient OR life-threatening prolonged seizures (>5 min) OR status epilepticus OR deep focal motor weakness OR diffuse cerebral edema on neuroimaging or clinical sign of elevated intracranial pressure
Investigations and non-pharmaceutical	ICE score and neuro signs q 4 h Evaluate and treat for other causes of AMS Delirium precautions	ICE score and neuro signs q 2–4 h Consider neurology consultation Evaluate and treat for other causes of AMS Delirium precautions Perform CT and consider MRI if not done in previous 24 h Consider EEG	ICE score and neuro signs q 2 h Intensive care unit transfer Evaluate and treat for other causes of AMS Delirium precautions Neurology consultation Perform CT and MRI imaging if not done in previous 24 h Consider EEG Consider lumbar puncture with pressure measurement	ICE score and neuro signs q 1 h Intensive care unit transfer Evaluate and treat for other causes of AMS Delirium precautions Neurology consultation Perform CT and MRI imaging if not done in previous 24 h Consider continuous EEG Consider lumbar puncture with pressure measurement
Treatment	Consider dexamethasone 10 mg IV × 1 Consider adding levetiracetam 500 mg PO BID for prophylaxis	Dexamethasone 10 mg IV q 6–12 h Levetiracetam 500 mg PO BID	Dexamethasone 10 mg IV q 6 h, if no improvement after 24 h, consider 20 mg IV q 6 h or methylprednisolone 1000 mg/kg IV q 12–24 h with anakinra 100 mg SC/IV q 12 h Levetiracetam 500 mg PO BID	Dexamethasone 10 mg IV q 6 h, if no improvement, consider high dose methylprednisolone 1000–2000 mg/kg IV q 12–24 h with anakinra 100 mg SC/IV q 12 h Levetiracetam 500 mg PO BID

ICE: Immune effector cell-associated encephalopathy; AMS: altered mental status; CT: computerized tomography scan; MRI: magnetic resonance imaging; EEG: electroencephalogram; PO: orally; SC: subcutaneously; IV: intravenously.

**Table 4:** Infection prophylaxis.

	Indication	Agent	Duration
Herpes simplex virus and varicella zoster virus	All patients	Valacyclovir 500 mg PO BID	Through treatment and until 6–12 months after the end
Pneumocystis jirovecii	All patients	Trimethoprim-sulfamethoxazole 800–160 mg one CO three times a week OR Atovaquone 1500 mg PO daily OR Pentamidine 300 mg inhaled q 4 weeks	Through treatment and until 6–12 months after the end
Bacterial	Optional, recommended if prolonged neutropenia, high infectious risk, or history of recurrent bacterial infections	Levofloxacin 500 mg PO daily OR Moxifloxacin 400 mg PO daily OR Doxycycline 200 mg PO daily	At least for the first 3 months of treatment and consider if persistent neutropenia or prolonged glucocorticoid use
Fungal	Consider in all patients Initiate if prolonged neutropenia	Fluconazole 400 mg PO daily	Until resolution of neutropenia
Immunoglobulins	Patients with IgG levels < 4 g/L	400 mg/kg every 2–4 weeks	Through treatment
G-CSF	Patients with neutrophils < 1.0 × 10^9^/L	Filgrastim 300–480 mcg SC daily or weekly	Target neutrophils > 1.0 × 10^9^/L

G-CSF: growth colony stimulating factor; BID: twice daily; SC: subcutaneously; PO: orally; CO: caplet.

**Table 5:** Management of cytopenias.

	Grade	Intervention
Anemia	Grade 3 (hemoglobin < 80 g/L) or symptoms	Continue treatment Consider transfusion
Neutropenia	Grade 3 (ANC 0.5–1.0 × 10^9^/L) without fever	Continue treatment Consider G-CSF use until ANC > 1.0 × 10^9^/L
	Grade 4 (ANC < 0.5 × 10^9^/L) or febrile neutropenia	Hold treatment until ANC > 1.0 × 10^9^/L Use G-CSF until ANC > 1.0 × 10^9^/L Consider extending dosing interval if desired response is achieved and myeloma is in good control Consider prophylactic G-CSF when restarting medication
Thrombocytopenia	Grade 4 (platelets < 25,000)	Hold treatment until platelets > 50,000
	Grade 3 (platelets 25,000–50,000) with bleeding	

ANC: absolute neutrophil count; G-CSF: growth colony stimulating factor.

## Data Availability

No new data were created or analyzed in this study. Data sharing is not applicable to this article.

## Abbreviations

The following abbreviations are used in this manuscript:

ASTCT	American Society for Transplantation and Cellular Therapy
CMV	Cytomegalovirus
CR	Complete response
DoR	Duration of response
EBV	Epstein–Barr virus
EEG	Electroencephalogram
EMA	European Medicines Agency
FDA	US Food and Drug Administration
HBV	Hepatitis B virus
HCV	Hepatitis C virus
HHV6	Human herpes virus 6
HSV	Herpes simplex virus
ICANS	Immune effector cell-associated neurotoxicity syndrome
ICE	Immune effector cell-associated encephalopathy
IMiD	Immunomodulator
IVIG	Intravenous immunoglobulins
MM	Multiple myeloma
MoAb	Monoclonal antibody
MRD	Minimal residual disease
ORR	Overall response rate
OS	Overall survival
PI	Proteasome inhibitor
PJP	Pneumocystis jirovecii
PR	Partial response
RRMM	Relapsed or refractory multiple myeloma
TCE	T-cell engager
VZV	Herpes zoster virus

## Appendix A

**Table A1:** Eligibility criteria for the virtual ward or outpatient administration.

Eligibility Criteria for the Virtual Ward		
Potential User Criteria		
	YES	NO
1. Subjects starting a treatment with a T-cell redirected therapy	◯	◯
2. Subjects residing or hosted in the territory of the delivering hospital and with the possibility of traveling to the designated site in less than an hour	◯	◯
3. Presence of a caregiver 24/7 during the step-up dosing, including the stay in virtual care ward	◯	◯
4. User is able to move independently by walking without support or with an assistive device	◯	◯
5. User and/or the caregiver reads and understands language used for communication	◯	◯
6. If needed, the caregiver is able to assist with communication between the user and the healthcare team	◯	◯
7. User is able to follow and adhere to the healthcare team’s instructions, including self-administration of medications	◯	◯
8. User and caregiver’s proficiency with devices enabling home hospitalization (including cellphones, tablets, and punctual monitoring device)	◯	◯
9. No anticipated infections or severe complications during the medical course or assessment	◯	◯
10. No drug or alcohol abuse by the user and the caregiver	◯	◯
11. User has given their consent	◯	◯

**Table A2:** Criteria for hospital admission for outpatient bispecific administration.

Criteria for Admission to Hospital From Virtual Ward		
Criteria for Admission		
	YES	NO
General condition deteriorating (clinical judgment)	◯	◯
New episode of fever	◯	◯
Hypotension or desaturation	◯	◯
Side effects of a medication requiring readmission	◯	◯
Neurotoxicity	◯	◯
Biological blood abnormalities	◯	◯
Transfusion needs, IV electrolytes, medical evaluation	◯	◯
Other:	◯	◯

**Table A3:** Criteria for virtual ward unit discharge.

Criteria for Virtual Ward Discharge		
Criteria for Discharge		
	YES	NO
48 h post-administration of day 8 cycle 1	◯	◯
Afebrile for at least 48 h	◯	◯
No hypotension or desaturation or confusion	◯	◯
No need for blood test within 72 h	◯	◯
No anticipated need for transfusion for 7 days	◯	◯

## Appendix B

**Table A4:** Immune effector cell-associated encephalopathy (ICE) score.

Orientation	4 points
Knows the year	1 point
Knows the month	1 point
Knows the city	1 point
Knows the name of the hospital	1 point
Obeys a simple command	1 point
Can write a legible sentence	1 point
Can identify three objects	3 points (1 point each)
Can count backwards from 100 by 10 s	1 point
